# The Prescription of Vitamin K Antagonists in a Very Old Population: A Cross-Sectional Study of 8696 Ambulatory Subjects Aged Over 85 Years

**DOI:** 10.3390/ijerph17186685

**Published:** 2020-09-14

**Authors:** Patrick Manckoundia, Clémentine Rosay, Didier Menu, Valentine Nuss, Anca-Maria Mihai, Jérémie Vovelle, Gilles Nuémi, Philippe d’Athis, Alain Putot, Jérémy Barben

**Affiliations:** 1 Hospital of Champmaillot, University Hospital, University of Burgundy, 21000 Dijon, France; rosayclm@gmail.com (C.R.); valentine.nuss@chu-dijon.fr (V.N.); anca-maria.mihai@chu-dijon.fr (A.-M.M.); jeremie.vovelle@chu-dijon.fr (J.V.); alain.putot@chu-dijon.fr (A.P.); jeremy.barben@chu-dijon.fr (J.B.); 2 UMR Inserm/U1093 Cognition, Action, Sensorimotor Plasticity, University of Burgundy and Franche Comté, 21000 Dijon, France; 3 Mutualité Sociale Agricole of Burgundy Franche Comté, 21000 Dijon, France; menu.didier@bourgogne.msa.fr; 4 Department of Medical Information, University Hospital, University of Burgundy, 21000 Dijon, France; gilles.nuemi@chu-dijon.fr (G.N.); philippe.athis@chu-dijon.fr (P.d.)

**Keywords:** aged 80 and over, anticoagulants, drug prescription, outpatients

## Abstract

We compared very elderly people taking vitamin K antagonists (VKA) and those not taking VKA (noVKA). Individuals were included in the noVKA group if there was no VKA on their reimbursed prescriptions during the study period. We also compared three subgroups, constituted by VKA type (fluindione, warfarin, or acenocoumarol). We included individuals aged over 85 years, affiliated to Mutualité Sociale Agricole of Burgundy, who were refunded for prescribed VKA in September 2017. The VKA and noVKA groups were compared in terms of demographic conditions, registered chronic diseases (RCD), number of drugs per prescription and cardiovascular medications. The three VKA subgroups were compared for the same items plus laboratory monitoring, novel and refill VKA prescriptions, and prescriber specialty. Of the 8696 included individuals, 1157 (13.30%) were prescribed VKA. Mean age was 90 years. The noVKA group had fewer women (53.67 vs 66.08%), more RCD (93.43 vs. 71.96%) and more drugs per prescription (6.65 vs. 5.18) than the VKA group (all *p* < 0.01). Except for direct oral anticoagulants and platelet aggregation inhibitors, the VKA group took significantly more cardiovascular medications. The most commonly prescribed VKA was fluindione (59.46%). Mean age was higher in the warfarin (90.42) than in the acenocoumarol (89.83) or fluindione (89.71) subgroups (*p* < 0.01). No differences were observed for sex (women were predominant) or RCD. 13% of subjects in this population had a VKA prescription. Fluindione was the most commonly prescribed VKA.

## 1. Introduction

Cardiovascular diseases are the most frequent comorbidities in older people, with a significant risk of death. Preventive or curative treatment of several cardiovascular diseases requires anticoagulation. Nonvalvular atrial fibrillation (AF) is among the most frequent cardiovascular diseases [1,2]. The prevalence of nonvalvular AF is 2% in the general population, but it rises to 13% after 75 years of age [3]. One main consequence of nonvalvular AF is embolic stroke [4]. Venous thromboembolism (VTE), including pulmonary embolism (PE), is the second main category of cardiovascular diseases [5,6] requiring anticoagulation [7,8].

There are two main types of oral anticoagulation treatments: Vitamin K antagonists (VKA) and direct oral anticoagulants (DOAC). VKA have been used since the 1940s, while DOAC were developed recently as an alternative to VKA. The main VKA on the market in France are fluindione, warfarin, and acenocoumarol. An assay of the international-normalized-ratio (INR) must be performed to monitor VKA treatment. The cumulative cost of VKA use (drug, laboratory monitoring, dose adjustment and iatrogenic complications) in France is estimated at 300 million euros per year [9].

The effectiveness of VKA in preventing thromboembolic events has been proven, especially in AF, and including in the elderly [9,10,11]. A recent review confirmed the benefit in prescribing warfarin for AF in people aged over 65 years [12]. However, when oral anticoagulation is initiated in a patient with AF who is eligible for a DOAC, the most recent guidelines from the European Society of Cardiology and European Heart Rhythm Association recommend using DOAC rather than VKA. The level of evidence for this recommendation is A [13,14]. The major risk associated with oral anticoagulants is bleeding. In 2007, the cumulative incidence of hemorrhagic risk in those treated with warfarin was 13.10% in individuals aged over 80 years, while it was only 4.70% below 80 years [15].

Although there are several studies on VKA, few have focused on all aspects of prescription, including prevalence, indications, follow-up and co-medications, in a very elderly population. The few studies found in the literature are limited to one aspect of prescription of VKA in the elderly or very elderly [16,17]. Thus, the main objective of our study was to describe the characteristics of a very elderly population (>85 years old) treated with VKA.

## 2. Material and Methods

### 2.1. Design

This cross-sectional study used data extracted from the existing database of a French regional agricultural health insurance agency (Mutualité Sociale Agricole de Bourgogne, Dijon, France); data were initially collected between 1 and 30 September, 2017. This study was conducted in accordance with the Declaration of Helsinki and French national standards. The Ethics Committee of our institution was consulted (2018-1002-PM). It approved this study which did not affect patient management.

### 2.2. Population

The population consisted of all Mutualité Sociale Agricole de Bourgogne-affiliated subjects older than 85 years living in Burgundy who had been refunded for a medically prescribed treatment during the study period. Two groups were constituted, one composed of subjects with a VKA prescription (VKAG) and another composed of subjects with no VKA prescription (noVKAG).

### 2.3. Collected Data

#### 2.3.1. Data Concerning All Subjects

For each subject, age and sex were collected. We also recorded registered chronic diseases (RCD) (according the International Classification of Diseases, 10th revision [18]) for which medical expenses are reimbursed by the French national health insurance agency, in accordance with French law. We also collected the number of drugs per prescription, concomitant cardiovascular medications including DOAC, heparins, other antithrombotics (including fondaparinux), platelet aggregation inhibitors, beta-blockers, central and peripheral adrenergic-blocking agents, angiotensin-conversion-enzyme inhibitors, angiotensin-receptor-blockers, calcium-channel-blockers, nitrate derivatives and other vasodilators, diuretics, cardiac glycosides including digitalis glycosides, other antiarrhythmic drugs, and hypolipidemic drugs.

For both groups, data were collected between the 1 and 30 September, 2017.

#### 2.3.2. Data Concerning Subjects With VKA

For the VKAG, we collected the type of VKA, the prescription duration, and the medical specialty of the prescribing physician. We also collected information regarding the prescription of laboratory investigations, i.e., complete blood cell count, natremia, kaliemia, serum creatinine, and blood urea nitrogen, aspartate aminotransferase rate, and alanine aminotransferase rate, γ-glutamyltranspeptidase rate, alkaline phosphatase rate, INR, prothrombin time, activated partial thromboplastin time, serum B_9_ and B_12_ vitamins, serum iron and serum ferritin. Finally, the indication(s) for VKA, AF, or VTE were deduced from declared RCD. We collected all data regarding coagulation tests, including INR monitoring, 3 months before to 3 months after the inclusion period, i.e., between June and December 2017. We selected this period in order not to overlook the INR testing that occurred closest to the subject’s inclusion (1 to 30 September 2017), knowing that depending on the balance of the AVK treatment, INR monitoring may be carried out daily to once a month or even less frequently.

Three VKA subgroups were constituted according to the prescribed molecule: Fluindione, warfarin or acenocoumarol.

### 2.4. Statistical Analysis

Categorical variables were described as numbers and percentages, while quantitative variables were described as means and standard deviations. A descriptive analysis was used to compare mean age, age ranges, sex, existence of one or more RCD, frequency of selected RCD, mean number of drugs per prescription, and concomitant prescriptions in the VKAG and the noVKAG.

Within the VKAG, a descriptive analysis was done to compare novel VKA prescriptions (initiation) with refill prescriptions (≥3 months) with regard to prescriber specialty. For a given subject, an AVK prescription was considered “novel” if, first, it was made within three months preceding the date of inclusion and, second, if no AVK prescription was found beyond three months before inclusion. This three-month period was chosen because in France, the maximum period of validity of a prescription for a drug from list 1, to which VKAs belong, is three months.

The three VKA subgroups were compared in terms of age, sex, frequency of selected RCD, type of RCD, mean number of drugs/prescription, cardiovascular medications, laboratory investigations, and prescriber specialty.

In bivariate analysis, data were compared using the chi-squared test or the Fisher test for categorical variables and the analysis of variance for quantitative variables. Statistical significance was set at *p* < 0.05. In order to study the association between VKA prescription and the presence of RCD, we performed a bivariate analysis using logistic regression, with the calculation of odds ratios adjusted to age and sex (aOR) and 95% confidence intervals (95% CI). Then, a multivariate analysis using stepwise logistic regression including variables with a threshold of 5% in bivariate analysis was performed to identify the RDCs independently associated with VKA prescription.

SAS^®^ 9.4 software (SAS^®^ 9.4, SAS Institute Inc., Cary, NC, USA) was used to conduct all statistical analyses.

## 3. Results

Altogether, we included 8696 individuals with a mean age (years) of 89.97 ± 3.32 (range 85–109); 64.43% were women and 35.57% were men.

In the total population, 15.82% (1376) of individuals had AF and 1.64% (143) had VTE.

### 3.1. Comparisons Between VKAG and noVKA

#### 3.1.1. Sociodemographic Characteristics, the Presence of AF and/or VTE, and the RCD Number

Table 1 shows sociodemographic characteristics (mean age, age ranges, sex), the rates of AF and VTE, and the mean number of RCDs in the VKAG and noVKAG. The VKAG included 1157 individuals and the noVKAG included 7539 individuals. Mean age (years) was 89.98 ± 3.16 in the VKAG, and 89.99 ± 3.34 in the noVKAG, with no significant difference (*p* = 0.912). There were more women than men in both groups, with significantly less female representation in the VKAG than in the noVKAG (*p* < 0.01). The rate of individuals with AF was significantly higher in the VKAG than in the noVKAG, 44.86% (519) and 11.37% (857), respectively (*p* < 0.0001). It was the same for VTE, 5.18% (60) and 1.10% (83), respectively (*p* < 0.0001).

#### 3.1.2. Comorbidities Deduced from RCDs

Table 2 compares RCDs in the VKAG and the noVKAG using bivariate and multivariate analyses (by logistic regression). In bivariate analysis, the VKA group had significantly more severe heart failure and/or arrhythmias, severe hypertension, and severe renal diseases. However, they had less chronic arterial occlusive diseases with ischemia, coronary artery disease, neurodegenerative and psychotic disorders, and cancer. After multivariate analysis, coronary artery disease, severe heart failure and/or arrhythmias, severe renal diseases, and psychotic disorders were the only significant factors (with other RCDs) for determining the VKA prescription or not.

#### 3.1.3. Prescriptions

The mean number of drugs/prescription was significantly higher in the VKAG than in noVKAG (6.65 ± 2.79 vs. 5.18 ± 2.82) (*p* < 0.01).

For each age range, the mean number of drugs/prescription was significantly higher in the VKAG (*p* < 0.01). In the VKAG, the mean number of drugs/prescription was 6.66 ± 2.78 for 86–90 years, 6.61 ± 2.84 for 91–95 years and 6.84 ± 2.67 for 96 years and older. In the noVKAG, the mean number of drugs/prescription increased up to the age of 95 years. The mean was 5.18 ± 2.86 for 86–90 years and 5.24 ± 2.82 for 91–95 years, decreasing to 4.97 ± 2.54 in individuals ≥96 years.

Women in the VKAG had significantly more prescribed drugs than women in the noVKAG (*p* = 0.02). The same trend was found for men (*p* = 0.02). The mean number of drugs/prescription for women was 6.80 ± 2.75 in the VKAG vs. 5.14 ± 2.78 in the noVKAG. The mean number of drugs/prescription for men was 6.49 ± 2.82 in the VKAG vs. 5.27 ± 2.91 in the noVKAG.

In the two groups, the mean number of drugs/prescription was significantly higher in the presence of one or more RCD than if there was no RCD (*p* = 0.04). In subjects with one or more RCD, the mean number of drugs/prescription was significantly higher in the VKAG than in the noVKAG (*p* = 0.04). In the VKAG, the mean number of drugs/prescription in individuals with one or more RCD was 6.70 ± 2.78 vs. 6.05 ± 2.86 when there was no RCD. In the noVKAG, the values were 5.56 ± 2.90 in individuals with one or more RCD vs. 4.23 ± 2.38 in those with no RCD.

#### 3.1.4. Cardiovascular Medications

Table 3 presents the prescriptions for cardiovascular medications in the VKAG and noVKAG.

DOAC and platelet aggregation inhibitors were significantly less prescribed in the VKAG than in the noVKAG. It was the opposite for heparins, beta-blockers, angiotensin-conversion-enzyme inhibitors, angiotensin-receptor-blockers, calcium-channel-blockers, nitrate derivatives, furosemide, spironolactone, digoxin, other antiarrhythmic drugs, and statins. In the noVKAG, 4348 (57.67%) subjects were not taking antithrombotics, 2445 (32.43%) were taking platelet aggregation inhibitors, 693 (9.19%) taking DOAC, 49 (0.65%) taking heparin, and 4 (0.05%) taking fondaparinux. The subgroup with no antithrombotic was significantly larger than each of the antithrombotic subgroups. The platelet aggregation inhibitors subgroup was significantly larger than the DOAC subgroup, while the DOAC subgroup was significantly larger than the heparin and fondaparinux subgroups (*p* < 0.01).

### 3.2. Specific Analyses in VKAG and Comparison Between the 3 VKA Subgroups

#### 3.2.1. Sociodemographic Characteristics and RCD

Fluindione (*N* = 688, 59.46%) was the most prescribed VKA, followed by warfarin (*N* = 431, 37.25%) and acenocoumarol (*N* = 38, 3.28%).

There were significant differences in mean age (years), respectively 89.71 ± 3.09 for the fluindione subgroup (range 85–102), 90.42 ± 3.22 for the warfarin subgroup (range 85–103) and 89.83 ± 3.30 for the acenocoumarol subgroup (range 85–98) (*p* < 0.01), and in age range between the 3 VKA subgroups (*p* = 0.016). Individuals aged 86–90 years made up 64.68% of the fluindione subgroup, 56.15% of the warfarin subgroup and 57.89% of the acenocoumarol subgroup. Subjects aged 91–95 years made up 29.21% of the fluindione subgroup, 36.43% of the warfarin subgroup and 36.84% of the acenocoumarol subgroup. Individuals aged 96–99 years were 5.96% of the fluindione subgroup, 6.96% of the warfarin subgroup and 5.26% of the acenocoumarol subgroup. Only three subjects were aged 100 or older (one in the fluindione subgroup and two in the warfarin subgroup).

Women were predominant in all three subgroups. They were 51.89% of the fluindione subgroup, 54.99% of the warfarin subgroup and 71.05% of the acenocoumarol subgroup. The difference between groups was not significant (*p* = 0.051).

Individuals with one or more RCD were not found significantly more often in one VKA subgroup than in another (*p* = 0.483). 81.25% of individuals of the fluindione subgroup had 1–3 RCDs and 12.35%, 4–8 RCDs. 81.67% of the warfarin subgroup had 1–3 RCDs and 11.14%, 4–8 RCDs. 89.47% of individuals in the acenocoumarol subgroup had 1–3 RCDs and 5.26% 4–8 RCDs.

#### 3.2.2. Indication VKA Prescription According to the RCD

In the VKAG, 44.86% of individuals were taking a VKA for AF and 5.18% for VTE.

AF and VTE were not significantly more common in any subgroup (for AF: 47.67% in the fluindione subgroup, 40.83% in the warfarin subgroup and 39.47% in the acenocoumarol subgroup, *p* = 0.065; for VTE: 4.94% in the fluindione subgroup, 5.10% in the warfarin subgroup and 10.53% in the acenocoumarol subgroup, *p* = 0.318), and neither were individuals with no RCD (6.25% in the fluindione subgroup, 7.19% in the warfarin subgroup and 5.26% in the acenocoumarol subgroup, *p* = 0.781).

#### 3.2.3. Mean Number of Drugs Per Prescription

Table 4 compares the mean number of drugs/prescription in the three VKA subgroups. There was no significant difference for the overall mean number of drugs/prescription (*p* = 0.108) or the mean number of drugs/prescription by age range (*p* = 0.734). The mean number of drugs/prescription was 6.81 ± 2.76 for one or more RCD vs. 5.51 ± 2.52 for no RCD in the fluindione subgroup, 6.45 ± 2.77 (≥1 RCD) vs. 6.81 ± 3.24 (no RCD) in the warfarin subgroup, and 7.39 ± 3.01 (≥1 RCD) vs. 6.00 ± 0.00 (no RCD) in the acenocoumarol subgroup. Because of insufficient number of individuals in one of the subgroups, it was not possible to calculate the *p* value.

#### 3.2.4. Cardiovascular Medications

Table 3 compares the cardiovascular medications prescribed in the three VKA subgroups.

The only three individuals in the VKAG who were prescribed DOAC were in the warfarin subgroup.

The individuals in the acenocoumarol subgroup had significantly more frequent prescriptions of platelet aggregation inhibitors than in the other subgroups.

Beta-blockers, angiotensin-conversion-enzyme inhibitors and other antiarrhythmic drugs were significantly more prescribed in the fluindione subgroup than in the warfarin subgroup and acenocoumarol subgroup.

Angiotensin-receptor-blockers, nitrate derivatives and spironolactone were significantly more prescribed in the acenocoumarol subgroup than in the fluindione subgroup and warfarin subgroup.

Digoxin and statins were significantly more prescribed in the acenocoumarol subgroup and fluindione subgroup than in the warfarin subgroup.

There was no significant difference between subgroups for calcium-channel-blockers or furosemide prescriptions.

#### 3.2.5. Laboratory Monitoring

Between June and December 2017, INR was measured in 97.75% of participants in the VKAG: 99.12% of individuals in the fluindione subgroup, 95.35% of individuals in the warfarin subgroup, and 100% of individuals in the acenocoumarol subgroup, with no significant difference between subgroups (*p* > 0.05)

#### 3.2.6. Comparison Between Novel and Refill VKA Prescriptions

Overall, 12.27% of participants had a novel VKA prescription and 87.72% had a refill (≥3 months).

Fluindione was the most frequently initiated VKA (73.24% (104/142) of novel prescriptions), followed by warfarin (20.42%), and acenocoumarol (6.34%), (*p* < 0.01). The result was similar for refill prescriptions: fluindione (57.53% of cases (584/1015)), then warfarin (39.61%), and acenocoumarol (2.86%) (*p* < 0.01).

Regardless the VKA subgroup, refill prescriptions, which represented 84.88% of fluindione prescriptions, 93.27% of warfarin prescriptions and 76.31% of acenocoumarol prescriptions, were significantly more frequent than novel prescriptions (*p* < 0.01).

#### 3.2.7. Prescriber Specialty

General practitioners were by far the most common prescribers of VKA (95.33% of cases), both for novel (92.25%) and refill prescriptions (95.76%). There was no significant difference in prescriber specialty between participants with novel and refill prescriptions (*p* = 0.085).

General practitioners were the main VKA prescribers in all subgroups, but the rate of prescribing general practitioners was significantly higher in the fluindione subgroup (96.80%) than in the warfarin subgroup (93.27%) or the acenocoumarol subgroup (92.10%) (*p* = 0.015).

## 4. Discussion

Given the iatrogenic nature of VKA, the constraints of laboratory monitoring, their low cost, and the ease of access to the antidote, we thought that it would be interesting to analyze the characteristics of very elderly people treated with VKA following the introduction of DOAC. Our study used data from a large ambulatory population in the region of Burgundy (France). Although there are studies on VKA, few of them focused on very elderly people or the characteristics that we considered in our work [12].

Our study shows that VKA are still often prescribed in elderly patients despite the emergence of DOAC. Indeed, we found that 13% of the individuals of the total population were still treated with VKA. This significant prescription rate could be explained by the lack of biological markers to control the effectiveness of DOAC and to minimize the risk of overdose.

Whatever the group, the majority were women, about 54% in VKAG and 66% in the noVKAG. This reflects the distribution of the French population.

Except for neuropsychiatric conditions (Alzheimer’s disease and severe neurocognitive disorders, Parkinson’s disease, psychotic disorders), ischemic peripheral or coronary artery diseases, and cancer, compared to VKAG, there were significantly fewer patients in the noVKAG with VTE (1.10% vs. 5.18%) or RCDs (severe heart failure, arrhythmia and/or AF (33.57% vs. 73.64%, of which for AF alone 11.37% vs. 44.86%), severe hypertension (22.16% vs. 24.88%), severe chronic nephropathy and/or primitive nephrotic syndrome (2.64% vs. 4.16%), and other RCDs (12.09% vs. 15.26%)). Multivariate analysis confirmed that ischemic peripheral artery disease and psychotic disorders were determining factors for non-VKA prescription, and on the contrary that severe heart failure, arrhythmia and/or AF, severe chronic nephropathy and/or primitive nephrotic syndrome, and other RCDs were determining factors for VKA prescription. The mean number of RCDs was significantly higher in the VKAG than in the noVKAG (2.21 vs. 1.94), and the average number of prescribed drugs was higher as well. The patients taking VKA were therefore more physically frail than those who were not. The high frequency of RCD in VKAG was particularly striking for cardiovascular diseases (heart failure and arrhythmia, hypertension). The risk factors for nonvalvular AF (e.g., hypertension, chronic heart failure, and coronary diseases) are common in frail populations, and polypathology is a marker of frailty [19,20]. Mostaza et al. found that compared with non-frail people, frail individuals had significantly more heart failure and coronary diseases [21]. In the elderly, Nguyen et al. showed that frailty in nonvalvular AF is an independent risk factor for death [22]. Another surprising result is the fact that 4/10 patients (38.12%) with AF and/or VTE in the total population were treated with VKA, even though the current recommendations leave little room for VKA [13,14]. Several explanations are possible. The first is related to increased risk of kidney failure with age. In our study, severe chronic nephropathies were more frequent in the VKAG than in noVKAG. Physicians would therefore probably have turned to a VKA, which is not contraindicated in case of kidney failure. On the contrary, DOACs must be used with caution because renal excretion may increase bleeding risk in people with kidney failure [23]. Hence, the French National Authority for Health (Haute Autorité de Santé; HAS) recommends VKA rather than DOAC in cases of severe renal failure [24]. Another explanation is the fact that, in clinical practice, physicians tend to be very cautious about prescribing new molecules in the very elderly population. They wait until there is some measure of hindsight in the non-elderly population before they feel confident about prescribing it in the very elderly population. This is probably why the VKAG contained more patients with RCDs, especially cardiovascular diseases, except for peripheral or coronary artery diseases, than the noVKAG. Finally, this study shows that 10% of patients in the noVKAG received an anticoagulant (DOAC, heparin or fondaparinux). It is likely that these drugs were prescribed for AF and/or VTE given that 12% of patients in the noVKAG had AF or VTE. The 2% difference (12% with a history of FA or VTE—10% on DOAC, heparin or fondaparinux) can be explained by the physician choosing not to start anticoagulation in view of an unfavorable benefit/risk ratio.

We found that there were more patients with ischemic peripheral or coronary artery diseases in the noVKAG than the VKAG. This can be explained by the fact that platelet aggregation inhibitors are indicated in these diseases and while VKA is not.

About 21% of participants taking VKA had cancer. A recent study found an increase of VTE recurrence in patients with cancer and treated in the long term with VKA compared to those receiving low-molecular-weight heparins. However, that study also found no increase of morbi-mortality in patients taking VKA or DOAC compared to those receiving low-molecular-weight heparin [25].

Severe neurocognitive disorders, including Alzheimer’s disease, were significantly less frequent in subjects taking VKA. Issues related to patient understanding and active participation probably influenced the prescribing physician’s choice more than the bleeding risk [26].

In our study, 7% of participants in the VKAG consumed platelet aggregation inhibitors. The association of VKA/platelet aggregation inhibitors is reserved for particular cases (both VKA indication and indication of platelet aggregation inhibitors), in particular because of the high risk of hemorrhage.

Whatever the age range and sex considered, the VKAG had significantly more prescribed drugs than the noVKAG. This can be explained by the fact that patients in the VKAG had significantly more RCDs. Moreover, the mean number of drugs/prescription was more than 5 in both groups, confirming the polypharmacy trend in the elderly. Drug–drug interactions and associated side effects are common in individuals who take multiple drugs [27]. In addition, polypharmacy is associated with nonadherence to treatment [28].

Concerning concomitant cardiovascular medications, beta-blockers, angiotensin-conversion-enzyme inhibitors, angiotensin-receptor-blockers, nitrate derivatives, diuretics, cardiac glycosides, other antiarrhythmic drugs, and hypolipidemic drugs (including statins) were more commonly prescribed in VKAG than in noVKAG. This result can be explained by the fact that cardiovascular diseases were more frequent in the VKAG [7,29,30,31]. A study showed a risk of overdose for phenprocoumon and acenocoumarol in cases of concomitant statins use [32]. These drug interactions raise questions regarding the role of statins in very elderly people treated with VKA.

Regarding the comparison of the three VKA subgroups, even if the statistical analysis indicates a significant difference for mean age, the differences are not clinically significant. We found that fluindione prescriptions were most common (about 60%) followed by warfarin, then distantly by acenocoumarol, both for novel and refill prescriptions. The distribution found in Burgundy is similar to that of the French population as a whole: the HAS reports that in France, currently, about 70% of patients treated with VKA are taking fluindione [33]. The predominant use of fluindione could be due to a lack of knowledge regarding the pharmacology of VKA, because warfarin has a greater half-life and therefore a greater stability. In addition, fluindione has a risk of immunoallergic complications [34]. In the rest of the world, warfarin is the most used VKA [33]. Given the 2018 recommendations from the HAS and The French National Agency for the Safety of Medicines and Health Products (Agence Nationale de Sécurité du Médicament et des produits de santé; ANSM), fluindione cannot be initiated but only continued in patients who are already taking this molecule [34]. Hence, warfarin and acenocoumarol should be preferred for VKA prescription.

In our study, 2.25% of the VKAG had no INR monitoring in the 3 months before or after a prescription for VKA. This is an obvious issue because of the associated major bleeding risk. While the retrospective access to limited patient records makes it difficult to interpret this finding, the vast majority of patients did have an INR follow-up.

This study demonstrates the primary role of general practitioners in prescribing VKA, for both novel (92% of prescriptions) and refill prescriptions (95%).

The results of our work would be difficult to apply to all types of populations. We chose to study a particular very old population who were affiliated to a French regional agricultural health insurance agency, but they did not share a common disease characteristic, such as a specific indication for VKA.

Our work has some limitations. First, the use of a retrospective cross-sectional study type exposes our work to the same drawbacks as a real world study, and the statistical relevance of our work could be debatable. The second bias is the indication for treatment. Indeed, indications were extrapolated from RCD because we did not have access to complete medical records. However, severe conditions are generally declared (RCD). Another limitation related to data collection is the fact that we were unable to access the entire medical history of included individuals. Thus, certain VTE events considered non-serious by the physician who made the diagnosis may have escaped a RCD declaration in the box that specifies “Illness leading to a serious medical condition”. This can explain why VKA indication was unknown for 50% of subjects in the VKAG. Indeed, 45% of patients had AF and 5% a VTE (Table 1), which are indications for VKA treatment. Thus, the VKA indication in the remaining 50% of subjects was probably mainly related to non-severe VTE. Finally, not taking into account self-medication also constitutes a bias. However, the evaluation of self-medication is difficult and often unreliable, regardless of the collection method, since it is often declarative.

## 5. Conclusions

Our study shows that VKA are still frequently used in the very elderly population despite the advent of DOAC. This trend may be associated with general practitioners taking a conservative approach to treatment in the elderly and the high cost of DOAC in comparison with VKA. The fact that some physicians may lack experience in prescribing anticoagulants could also partly explain the results of our study.

Unsurprisingly, polypathology and polypharmacy was frequent in the participants taking VKA.

Future studies comparing VKA and DOAC prescriptions in the very elderly and ambulatory population would make it possible to elucidate the criteria for prescribing, in particular for general practitioners, one oral anticoagulant rather than another.

## Figures and Tables

**Table 1:** Comparison of demographic conditions (age and sex), rates of atrial fibrillation (AF) and venous thromboembolism (VTE), and mean number of registered chronic diseases (RCD) in subjects prescribed or not prescribed vitamin K antagonists.

Parameter		VKAG (*N* = 1157)	noVKAG (*N* = 7539)	*p*
Age (years)	Mean ± SD	89.98 ± 3.16	89.99 ± 3.33	0.912
	Range	85–03	85–109	
		% (*N*)	% (*N*)	0.267
	86–90	61.28 (709)	62.06 (4679)	
	91–95	32.15 (372)	30.31 (2285)	
	96–100	6.31 (73)	7.04 (531)	
	>100	0.26 (3)	0.58 (44)	
Sex	Women	53.67 (621)	66.08 (4982)	<0.01
	Men	46.33 (536)	33.92 (2557)	
Atrial fibrillation		44.86 (519)	11.37 (857)	<0.0001
Venous thromboembolism		5.18 (60)	1.10 (83)	<0.0001
Mean number of RCD		2.21 ± 1.09	1.94 ± 1.04	<0.01

VKAG: vitamin K antagonist group; noVKAG: no vitamin K antagonist group; SD: standard deviation; N: number; RCD: registered chronic diseases.

**Table 2:** Comparison of registered chronic diseases in subjects prescribed or not prescribed vitamin K antagonists, using bivariate and multivariate analyses using logistic regression.

Parameter	VKAG	noVKAG	Bivariate Analysis		Multivariate Analysis	
	% (*N*)	% (*N*)	aOR (95% CI)	*p*	aOR (95% CI)	*p*
noRCD	6.57 (76)	28.04 (2114)		<0.01		<0.01
RCD	93.43 (1081)	71.96 (5425)				
Disabling stroke	7.68 (83)	7.58 (411)	1.01 (0.79–1.29)	0.945		
Bone marrow failure and/or other chronic cytopenias	0.19 (2)	0.41 (22)	0.45 (0.11–1.93)	0.284		
CAOD with ischemic manifestations	8.14 (88)	9.90 (537)	0.76 (0.60–0.97)	0.026	0.76 (0.59–0.97)	0.030
Severe heart failure, arrhythmia, and/or atrial fibrillation	73.64 (796)	33.57 (1821)	5.48 (4.73–6.35)	<0.01	5.50 (4.74–6.37)	<0.01
Diabetes mellitus (types 1 et 2)	19.24 (208)	21.44 (1163)	0.87 (0.73–1.02)	0.089		
Neuropathy and/or myopathy and/or epilepsy	0.83 (9)	0.98 (53)	0.88 (0.43–1.79)	0.722		
Severe hypertension	24.88 (269)	22.16 (1202)	1.19 (1.02–1.39)	0.025		
Coronary artery disease	19.89 (215)	21.70 (1177)	0.84 (0.71–0.99)	0.037		
Severe chronic respiratory failure	5.55 (60)	4.15 (225)	1.27 (0.95–1.71)	0.107		
Alzheimer’s disease and severe neurocognitive disorders	9.25 (100)	14.93 (810)	0.61 (0.49–0.76)	<0.01		
Parkinson disease	1.76 (19)	2.84 (154)	0.59 (0.37–0.96)	0.035		
Severe chronic nephropathy and/or PNS	4.16 (45)	2.64 (143)	1.54 (1.09–2.17)	0.014	1.45 (1.01–2.09)	0.028
Polyarthritis nodosa and/or SLE and/or systemic scleroderma	0.46 (5)	0.76 (41)	0.62 (0.24–1.56)	0.308		
Severe rheumatoid arthritis	1.29 (14)	2.10 (114)	0.65 (0.37–1.13)	0.126		
Psychotic disorders	2.87 (31)	5.49 (288)	0.54 (0.37–0.79)	<0.01	0.64 (0.43–0.94)	0.024
Cancer and/or hematologic malignancy	20.91 (226)	24.98 (1355)	0.74 (0.63–0.87)	<0.01		
Other RCDs	15.26 (165)	12.09 (656)	1.36 (1.13–1.64)	<0.01	1.41 (1.16–1.72)	<0.01
Illness leading to a serious medical condition	2.31 (25)	3.61 (196)	0.67 (0.44–1.02)	0.062		
Non-exempting RCDs	1.76 (19)	2.03 (110)	0.89 (0.54–1.45)	0.630		

VKAG: vitamin K antagonist group, noVKAG, no vitamin K antagonist group, N: number, aOR: odds ratios adjusted, CI: confidence intervals, RCD: registered chronic diseases, CAOD: chronic arterial occlusive diseases, PNS: primitive nephrotic syndrome, SLE: systemic lupus erythematosus.

**Table 3:** Comparison of cardiovascular medications in subjects prescribed or not prescribed vitamin K antagonists on the one hand, and the three subgroups of vitamin K antagonists on the other hand.

Drugs	VKAG (*N* = 1157)	noVKAG (*N* = 7539)	*p* *	Fluindione (*N* = 688)	Warfarin (*N* = 431)	Acenocoumarol (*N* = 38)	*p* **
	% (*N*)	% (*N*)		% (*N*)	% (*N*)	% (*N*)	
Direct oral anticoagulants	0.26 (3)	9.19 (693)	<0.01	0 (0)	0.70 (3)	0 (0)	<0.01
Heparins	1.38 (16)	0.65 (49)	0.01	0.87 (6)	2.32 (10)	0 (0)	0.649
Fondaparinux	0.09 (1)	0.05 (4)	***	0.14 (1)	0 (0)	0 (0)	0.053
Platelet aggregation inhibitors	7.00 (81)	32.43 (2445)	<0.01	7.27 (50)	6.50 (28)	7.89 (3)	<0.01
Beta-blockers	38.12 (441)	24.71 (1863)	<0.01	38.37 (264)	37.82 (163)	36.84 (14)	<0.01
Central ABA	1.90 (22)	2.56 (193)	0.214	1.89 (13)	2.09 (9)	0 (0)	***
Peripheral ABA	1.82 (21)	1.84 (139)	0.960	2.03 (14)	1.39 (6)	2.63 (1)	***
ACE inhibitors	19.62 (227)	15.07 (1136)	<0.01	21.22 (146)	18.33 (79)	5.26 (2)	<0.01
Angiotensin-receptor-blockers	14.26 (165)	11.22 (846)	<0.01	14.10 (97)	13.92 (60)	21.05 (8)	0.013
Calcium-channel-blockers	22.13 (256)	19.18 (1446)	0.021	22.53 (155)	22.04 (95)	15.79 (6)	0.087
Nitrate derivatives	7.00 (81)	5.33 (402)	<0.01	7.56 (52)	5.80 (25)	10.53 (4)	0.049
Other vasodilators	3.46 (40)	2.48 (187)	0.06	4.65 (32)	1.39 (6)	5.26 (2)	***
Furosemide ^$^	54.45 (630)	25.79 (1944)	<0.01	54.07 (372)	53.83 (232)	68.42 (26)	0.212
Spironolactone ^$^	6.83 (79)	3.32 (250)	<0.01	6.69 (46)	6.03 (26)	18.42 (7)	0.014
Digoxin	14.52 (168)	3.18 (240)	<0.01	15.55 (107)	12.76 (55)	15.79 (6)	<0.01
Other antiarrhythmic drugs	9.51 (110)	4.44 (335)	<0.01	9.88 (68)	9.28 (40)	5.26 (2)	<0.01
Statins	21.43 (248)	17.80 (1342)	<0.01	23.11 (159)	18.56 (80)	23.68 (9)	0.005
Fibrates	2.51 (29)	2.82 (213)	0.604	2.91 (20)	1.86 (8)	2.63 (1)	***
Bile acid sequestrants	0.09 (1)	0.04 (3)	***	0 (0)	0.232 (1)	0 (0)	***
Other hypolipidemic drugs	0.78 (9)	0.48 (36)	0.268	0.58 (4)	0.93 (4)	2.63 (1)	***

VKAG: vitamin K antagonist group, noVKAG: no vitamin K antagonist group, N: number, ABA: adrenergic-blocking agents, ACE: angiotensin-conversion-enzyme; ^$^: Only these two diuretics are presented because the others were too rare to lead to a meaningful analysis; *: Comparison between the VKAG and noVKAG; **: Comparison between the 3 VKA subgroups; ***: Insufficient number to calculate the *p* value.

**Table 4:** Comparison of number of drugs per prescription (global, according to the gender, age range, and registered chronic diseases) in the three subgroups of vitamin K antagonists.

Parameter		VKA Subgroup			*p*
		Fluindione (*N* = 688)	Warfarin (*N* = 431)	Acenocoumarol (*N* = 38)	
NDP		Mean ± SD	Mean ± SD	Mean ± SD	
Per subgroup		6.73 ± 2.76	6.48 ± 2.81	7.32 ± 2.94	0.108
According to the sex	Women	6.79 ± 2.65	6.79 ± 2.87	6.89 ± 2.97	0.047
	Men	6.66 ± 2.88	6.09 ± 2.67	8.36 ± 2.73	
According to the age range (years)	86–90	6.68 ± 2.72	6.54 ± 2.83	7.55 ± 3.29	0.734
	91–95	6.8 ± 2.92	6.32 ± 2.73	7 ± 2.51	
	96–100	7 ± 2.4	6.63 ± 3.08	7 ± 2.83	
	>100	4 *	8 ± 1.41	0	
According to the RCD	≥1 RCD	6.81 ± 2.76	6.45 ± 2.77	7.39 ± 3.01	***
	No RCD	5.51 ± 2.52	6.81 ± 3.24	6 **	

VKA: vitamin K antagonist, N: number, NDP: number of drugs per prescription, SD: standard deviation, RCD: registered chronic diseases.*: Only 1 subject; **: 2 subjects; ***: Insufficient number to calculate the *p* value.
